# Correlation between Identification of β-Lactamase Resistance Genes and Antimicrobial Susceptibility Profiles in Gram-Negative Bacteria: a Laboratory Data Analysis

**DOI:** 10.1128/spectrum.01485-21

**Published:** 2022-03-07

**Authors:** Ammara Mushtaq, Rachel Chasan, Michael D. Nowak, Meenakshi Rana, Sahrish Ilyas, Alberto E. Paniz-Mondolfi, Emilia M. Sordillo, Gopi Patel, Melissa R. Gitman

**Affiliations:** a Division of Infectious Disease, Department of Medicine, Icahn School of Medicine at Mount Sinai, New York, New York, USA; b Department of Pathology, Molecular, and Cell Based Medicine, Icahn School of Medicine at Mount Sinai, New York, New York, USA; Universidad de Buenos Aires, Facultad de Farmacia y Bioquimica

**Keywords:** CTX-M, Enterobacterales, ceftriaxone, antimicrobial resistance, Gram-negative bacteria, rapid molecular diagnostics

## Abstract

We reported the frequency of resistance gene detection in Gram-negative blood culture isolates and correlated these findings with corresponding antibiograms. Data were obtained from 1045 isolates tested on the GenMark Dx ePlex Blood Culture Identification Gram-Negative Panels at the Mount Sinai Hospital Clinical Microbiology Laboratory in New York from March 2019 to February 2021. Susceptibilities were performed using Vitek 2 (bioMérieux Clinical Diagnostics) or Microscan (Beckman Coulter Inc.). *bla*_CTX-M_ was detected in 26.4% Klebsiella pneumoniae, 23.5% Escherichia coli, and 16.4% Proteus mirabilis isolates. As would be expected, both *bla*_CTX-M_ and *bla*_CTX-M_ negative isolates were likely to be susceptible to newer agents while *bla*_CTX-M_ positive isolates were more likely to be resistant to earlier generations of beta-lactam antibiotics. 3/204 *bla*_CTX-M_-positive isolates were found to be ceftriaxone-susceptible. Conversely, 2.8% ceftriaxone nonsusceptible strains were negative for all β-lactamase genes on the ePlex BCID-GN panel, including *bla*_CTX-M_. The prevalence of CTX-M-producing Enterobacterales remains high in the United States. A small number of *bla*_CTX-M_-positive isolates were susceptible to ceftriaxone, and a small number of ceftriaxone nonsusceptible isolates were negative for *bla*_CTX-M_. Further studies are needed to determine the optimal management when an isolate is phenotypically susceptible to ceftriaxone, but *bla*_CTX-M_ is detected.

**IMPORTANCE** There is limited literature on corresponding results obtained from rapid molecular diagnostics with the antibiotic susceptibility profile. We reported a correlation between the results obtained from ePlex and the antibiograms against a large collection of Gram-negative bacteria. We reported that there can be a discrepancy in a small number of cases, but the clinical significance of that is unknown.

## INTRODUCTION

The incidence of infections caused by extended-spectrum β-lactamase (ESBL)-containing Enterobacterales increased by more than 50% between 2012 and 2017 (1). ESBLs are active against expanded spectrum cephalosporins and aztreonam, but not against cephamycins such as cefoxitin. Among all ESBLs, CTX-Ms are the most identified enzymes in the United States and, therefore, have a significant epidemiological and clinical impact (2).

Current estimates of the prevalence of ESBLs in the United States are based on phenotypic ESBL tests or ceftriaxone nonsusceptibility rather than direct molecular detection (3). The use of ceftriaxone nonsusceptibility as a surrogate marker for ESBL prevalence may overestimate the prevalence of ESBL because mechanisms other than ESBL production, such as plasmid-mediated ampC (p-ampC) β-lactamases, also cause ceftriaxone resistance. In addition, phenotypic ESBL production testing is done routinely only for Escherichia coli, Klebsiella pneumoniae, Klebsiella oxytoca, and Proteus mirabilis (4). The use of rapid molecular diagnostic (RMD) tests such as GenMark ePlex BCID-GN panel allows surveillance of CTX-M ESBL and common carbapenemases at a molecular level in Gram-negative bacteria. RMD tests support a mechanistic approach for the selection of empirical antibiotics that may directly impact patient outcomes. For example, in a case with E. cloacae complex bacteremias, cefepime was an appropriate treatment for a ceftriaxone-resistant, cefepime-susceptible isolate that was ESBL-negative, whereas cefepime use has been associated with increased mortality for such cases if the isolate is ESBL-positive (5). Genotypic prediction of susceptibility profiles has been shown to improve outcomes, especially when combined with antimicrobial stewardship initiatives (6).

The GenMark Dx ePlex Blood Culture Identification system provides a rapid turnaround time of 1.5 h and is independent of organism growth (6). There is a paucity of data regarding the incidence of bacterial isolates with a mismatch between the molecular and phenotypic susceptibility profiles. Such genotypic-phenotypic discordance can lead to inappropriate escalation or deescalation of antibiotics (6). There is scarce guidance on the appropriate management of such discordant cases.

Our primary objective was to study the prevalence of *bla*_CTX-M_, *bla*_KPC_, *bla*_OXA-48-like/23_, *bla*_NDM_, *bla*_IMP_, and *bla*_VIM_ among Gram-negative clinical blood culture isolates and concordance with phenotypic susceptibility profiles at a clinical microbiology laboratory serving a large health care system in New York City. To our knowledge, this is the first large report addressing the issue of genotypic-phenotypic discordance in the era of RMD testing.

## RESULTS

A total of 1045 Gram-negative isolates were studied. The distribution of the Gram-negative isolates is shown in Table 1. E. coli followed by K. pneumoniae were the most frequently observed isolates. There was 100% agreement between identification obtained by matrix-assisted laser desorption ionization-time of flight mass spectrometry (MALDI-ToF MS) and ePlex at the genus level. The identification of Klebsiella aerogenes by MALDI-ToF MS and Enterobacter non-cloacae complex by ePlex was considered to agree.

**TABLE 1:** Distribution of Gram-negative bacteria identified by MALDI-ToF and ePlex from March 2019 to February 2021

ID by MALDI-ToF MS	ID by ePlex	No. of isolates
Escherichia coli	Escherichia coli	496
Klebsiella pneumoniae complex	Klebsiella pneumoniae (*n* = 254) Klebsiella pneumoniae and Klebsiella oxytoca (*n* = 2) Klebsiella oxytoca (*n* = 1)	257
Pseudomonas aeruginosa	Pseudomonas aeruginosa	74
Proteus mirabilis	Proteus mirabilis	73
Enterobacter cloacae complex	Enterobacter cloacae	44
Serratia marcescens	Serratia marcescens	28
Klebsiella aerogenes	Enterobacter (noncloacae complex)	15
Citrobacter freundii	Citrobacter sp.	14
Klebsiella oxytoca	Klebsiella oxytoca	11
Morganella morganii	Morganella morganii	9
Acinetobacter baumannii	Acinetobacter baumannii	8
Salmonella sp.	Salmonella sp.	8
Citrobacter koseri	Citrobacter sp.	6
Acinetobacter nosocomialis	Acinetobacter baumannii	1
Proteus vulgaris	Proteus sp.	1

The total prevalence of *bla*_CTX-M_ in Gram-negative isolates was 19.5%. *bla*_CTX-M_ was detected in 26.4% K. pneumoniae (*n* = 68/257), 23.5% E. coli (*n* = 117/496), and 16.4% P. mirabilis (*n* = 12/73). It was also detected in 1/44 Enterobacter cloacae complex, 3/14 Citrobacter freundii, 1/15 Klebsiella aerogenes, 1/9 Morganella morganii, and 1/8 Salmonella sp.

*bla*_KPC_ was detected in 1.1% (*n* = 3) of K. pneumoniae isolates. *bla*_KPC_ was also detected in three additional isolates of E. cloacae complex, E. coli, and K. oxytoca. *bla*_NDM_ was detected in one isolate of K. pneumoniae and one isolate of E. coli. A K. pneumoniae isolate had both *bla*_KPC_ and *bla*_CTX-M_, and another K. pneumoniae isolate had *bla*_KPC_ and *bla*_NDM._

The *bla*_NDM_-positive E. coli isolate was resistant to all tested β-lactams, β-lactams with β-lactamase inhibitors (BLBLI), and fluoroquinolones but was susceptible to amikacin, gentamicin, tigecycline, tobramycin, and trimethoprim-sulfamethoxazole (TMP-SMX).

The *bla*_NDM_/*bla*_KPC-_positive K. pneumoniae had a broader resistance profile and was nonsusceptible to all tested β-lactams, BLBLI, fluoroquinolones, tobramycin, gentamicin, and TMP-SMX; and susceptible to amikacin and tigecycline. Among the remaining 5 *bla*_KPC_-positive isolates (2 K. pneumoniae, 1 K. oxytoca, 1 E. coli, and 1 E. cloacae complex), all isolates were nonsusceptible to ceftazidime, ceftriaxone, and imipenem, and 1/5 to amikacin, 3/3 ampicillin-sulbactam, 4/4 ampicillin, 1/1 aztreonam, 1/1 cefazolin, 1/2 cefepime, 2/2 ceftolozane-tazobactam, 1/1 cefuroxime, 4/5 ciprofloxacin, 1/1 ertapenem, 3/5 gentamicin, 3/4 levofloxacin, 2/2 meropenem, 4/4 piperacillin-tazobactam, 0/1 tetracycline, 1/5 tigecycline, 5/5 tobramycin, and 3/5 TMP-SMX. One isolate of K. oxytoca was dose-dependent susceptible to cefepime.

*bla*_OXA-48-like/23_ gene was detected in one isolate of K. pneumoniae. This isolate tested as susceptible to ampicillin-sulbactam, cefepime, ceftazidime, ceftriaxone, ciprofloxacin, ertapenem, gentamicin, imipenem, levofloxacin, piperacillin-tazobactam, tobramycin and TMP-SMX.

No β-lactam resistance genes were detected in Acinetobacter baumannii, Acinetobacter nosocomialis, Citrobacter koseri, Serratia marcescens, and Pseudomonas aeruginosa by ePlex.

Of the 204 *bla*_CTX-M_-positive isolates, three were found to be ceftriaxone-susceptible (C. freundii, E. coli, and K. pneumoniae), 143 were ceftriaxone non-susceptible, and ceftriaxone susceptibility was not performed in 58 cases (likely due to serial blood cultures being positive). The differential susceptibility profiles of *bla*_CTX-M_-positive isolates, compared to those of *bla*_CTX-M_-negative isolates of E. coli, K. pneumoniae, and P. mirabilis are shown in Table 2. Isolates that had a resistance gene other than *bla*_CTX-M_ identified were excluded from this analysis. One K. pneumoniae positive for *bla*_CTX-M_ and *bla*_KPC_ was excluded from the ‘*bla*_CTX-M_-positive group’. In the ‘*bla*_CTX-M_-negative group’ (*n* = 629), the following five isolates were excluded: two *bla*_KPC_-positive isolates (E. coli and K. pneumoniae), two *bla*_NDM_-positive isolates (E. coli and K. pneumoniae), and one *bla*_OXA-48-like/23_-positive K. pneumoniae. Both *bla*_CTX-M_-positive and negative isolates had a high likelihood of susceptibility to amikacin, ceftazidime-avibactam, ceftolozane-tazobactam, ertapenem, imipenem (except Proteus sp.), meropenem, and tigecycline. Compared to *bla*_CTX-M_-negative isolates, relatively low susceptibilities were seen in *bla*_CTX-M_-positive isolates for ampicillin-sulbactam, gentamicin, piperacillin-tazobactam, tobramycin, and TMP-SMX, whereas a larger differential was seen in susceptibilities for ampicillin (likely due to penicillinases in the case of K. pneumoniae), aztreonam, cephalosporins, and fluoroquinolones (Table 2). The use of fluoroquinolones continues to be rising, as they are good oral agents with high bioavailability and cover P. aeruginosa. Our study observed a high frequency of fluoroquinolone resistance in *bla*_CTX-M_ positive versus *bla*_CTX-M_ negative. While CTX-M does not hydrolyze non-β-lactams like fluoroquinolones, the presence of ESBL often coexists with other mechanisms of resistance, and hence, confers a multidrug-resistant profile (7).

**TABLE 2:** Differential antibiotic susceptibility profile of *bla*_CTX-M_ positive *versus* negative K. pneumoniae, E. coli, and P. mirabilis

Antibiotics	*bla*_CTX-M_ positive (*n* = 196) (% [susceptible isolates/ total no. of isolates])	*bla*_CTX-M_ negative (*n*= 624) (% [susceptible isolates/ total no. of isolates])	Chi-square statistic (*P* value)
Ampicillin	0.7 (1/139)	34.8 (156/447)	63.1 (<0.00001)
Ampicillin-sulbactam	41.1 (21/51)	69 (306/443)	15.9 (0.00006)
Piperacillin-tazobactam	78 (39/50)	94.7 (419/442)	19.69 (<0.00001)
Cefazolin	0 (0/35)	72.2 (78/108)	55.6 (<0.00001)
Cefuroxime	2.7 (1/36)	93.2 (110/118)	112.11 (<0.00001)
Ceftriaxone	1.4 (2/139)	98.6 (441/447)	543.22 (<0.00001)
Ceftazidime	34.5 (48/139)	97.3 (435/447)	288.4 (<0.00001)
Cefepime^*a*^	54 (74/137)	99.3 (444/447)	253.7 (<0.00001)
Ceftazidime-Avibactam	97.6 (42/43)	100 (117/117)	2.73 (0.09)
Ceftolozane-Tazobactam	97.2 (35/36)	100 (117/117)	3.27 (0.07)
Aztreonam	13.8 (5/36)	91.8 (112/122)	87.8 (<0.00001)
Ertapenem	97 (133/137)	100 (447/447)	17.83 (0.000024)
Imipenem^*b*^	96.1 (125/130)	100 (411/411)	15.95 (0.00006)
Meropenem	97.2 (36/37)	100 (118/118)	2.72 (0.09)
Amikacin	96.4 (134/139)	99.5 (445/447)	8.91 (0.002)
Gentamicin	66.1 (92/139)	93.2 (417/447)	68.23 (<0.00001)
Tobramycin	55.3 (77/139)	94.4 (422/447)	127.63 (<0.00001)
Tetracycline	36.3 (12/33)	63.5 (75/118)	7.81 (0.005)
Tigecycline	87.8 (36/41)	99.1 (111/112)	10.17 (0.001)
Ciprofloxacin	24.4 (34/139)	85.5 (380/444)	192.14 (<0.00001)
Levofloxacin	25.1 (35/139)	85.8 (381/444)	190.39 (<0.00001)
Trimethoprim-sulfamethoxazole	29.4 (41/139)	77.1 (345/447)	107.24 (<0.00001)

a Includes 23 dose-dependent susceptible isolates in *bla*_CTX-M_ positive, and 3 in the *bla*_CTX-M_ negative group.

b Excludes P. mirabilis.

A *bla*_CTX-M_ positive K. pneumoniae resistant to ceftolozane-tazobactam was noted. In addition, four more *bla*_CTX-M_ positive isolates were aztreonam-susceptible, but ceftriaxone resistant (3 P. mirabilis and 1 E. coli).

The antibiogram of the three *bla*_CTX-M_-positive ceftriaxone-susceptible isolates is shown in Table 3. These isolates were susceptible to most antibiotics, except the E. coli isolate that was resistant to fluoroquinolones and TMP-SMX and the expected ampicillin resistance in K. pneumoniae.

**TABLE 3:** Antimicrobial susceptibility profile of *bla*_CTX-M_-positive ceftriaxone-susceptible isolates

Antibiotic	No. of susceptible isolates/total no. of isolates tested
Amikacin	3/3
Ampicillin-sulbactam	2/2
Ampicillin	1/2
Aztreonam	1/1
Cefepime	3/3
Ceftazidime	3/3
Ceftazidime-avibactam	1/1
Ceftolozane-tazobactam	1/1
Ceftriaxone	3/3
Cefuroxime	1/1
Ciprofloxacin	2/3
Ertapenem	3/3
Gentamicin	3/3
Imipenem	3/3
Levofloxacin	2/3
Meropenem	1/1
Piperacillin-tazobactam	3/3
Tetracycline	1/1
Tigecycline	1/1
Tobramycin	3/3
Trimethoprim-sulfamethoxazole	2/3

On the other hand, 2.87% (*n* = 30/1045) isolates were negative for *bla*_CTX-M_ and other resistance genes that were investigated and were ceftriaxone non-susceptible. This was observed most with E. cloacae complex (*n* = 12), but also with Enterobacter noncloacae (Klebsiella aerogenes, *n* = 4), E. coli (*n* = 3), S. marcescens (*n* = 3), C. freundii (*n* = 2), M. morganii (*n* = 2), P. mirabilis (*n* = 2), K. oxytoca (*n* = 1), and K. pneumoniae (*n* = 1).

## DISCUSSION

Our results showed that the correlation of susceptibility profile with the detection of resistance genes by ePlex varied by the gene detected. In two cases of identification of *bla*_NDM_, the phenotypic profile correlated 100%. Only one isolate was positive for *bla*_OXA-48-like/23_, which was highly susceptible to all tested β-lactams, an expected finding (8). Among the six *bla*_KPC_ positive isolates, the only discrepancy was a dose-dependent susceptibility to cefepime in one case. For *bla*_CTX-M_ positivity, overall, results correlated to ceftriaxone nonsuceptibility. However, three isolates were ceftriaxone susceptible, and an additional four were aztreonam susceptible (but ceftriaxone resistant). Our data did not have any positives for *bla*_VIM_ and *bla*_IMP_. Therefore, a correlation could not be established for these genes. Our study showed that a small percentage of isolates that are *bla*_CTX-M_-positive by molecular testing were phenotypically susceptible to ceftriaxone. There are a few plausible explanations for this finding. It can be hypothesized that certain variants of CTX-M are less fit, for example, because of a minor mutational change, such as a single base change, eliminating the activity of the resulting protein, and not conferring resistance to β-lactams. CTX-M-93, a CTX-M variant, is one such example. It has been found to confer higher MICs of ceftazidime, thus resulting in increased ceftazidime hydrolysis and decreased MICs of other cephalosporins, such as cefotaxime and penicillins, characterized by lacking significant penicillin hydrolysis (9). CTX-M-71, another CTX-M variant characterized by one amino acid substitution from glycine to cysteine at position 238, resulted in the decreased hydrolytic activity of the β-lactamase for cefotaxime, ceftazidime, and cefepime (10). This phenomenon of a minor mutational change in a single base resulting in a nonfunctional enzyme was recently reported for KPC-2 (11). A C-to-T mismatch resulted in a change in a codon encoding an amino acid into a stop codon. This led to the production of a truncated, non-functional KPC (1). For CTX-M, more than 250 allelic variants have been described, and they are clustered into five groups (12). Up to 32% of amino-acid divergence is observed among different groups of CTX-M, and <5% amino acid sequence difference is observed within each group (2). However, the differential susceptibility of these groups and variants to different β-lactams is largely unknown. Several amino acids in the CTX-M enzyme are functionally important for its cefotaxime-preferred hydrolytic activity, which include the Asn104, Ser237, Asp240, and Arg276 amino acids in the β3-strand and the Ω-loop (13). Owing to this allotypic diversity and variety of genetic content of CTX-M, there is likely evolution in substrate specificity because of point mutations, which occurs with TEM and SHV β-lactamases (2). Classically, CTX-M enzymes hydrolyze ceftriaxone and cefotaxime, but not ceftazidime. Certain mutants of CTX-M, however, have enhanced ceftazidimase activity (e.g., Asp240Gly and Pro167Ser variants) (2). Another plausible explanation that merits investigation is that ceftriaxone-susceptible isolates have a scarce amount of CTX-M enzyme which was detected by the molecular test but was not enough to confer resistance to ceftriaxone. Further understanding of such discordant cases, whether because of differential substrate specificity, the quantity of the enzyme present, or accuracy of the genotypic assay, can aid in the development of novel antibiotics and improvement of diagnostic tests, and support antimicrobial stewardship. The three *bla*_CTX-M_-positive, ceftriaxone-susceptible isolates in this study have retained high susceptibility to all antibiotics, including β-lactams. Therefore, it appears very likely that the *bla*_CTX-M_ variant presents either (i) has a mutation that renders it unfit so that it does not confer resistance, (ii) a regulatory mutation reduces the amount of enzyme produced, or (iii) there was a false-positive result in the ePlex assay. To confirm the ePlex results, it would also be important to retest discordant isolates with the ePlex. Further molecular characterization may be needed to better understand the reason behind the genotypic to phenotypic discordance; however, this testing is beyond the scope of this study. On the other hand, 2.8% of the isolates were ceftriaxone-resistant and negative for the genes included on the ePlex BCID GN panel. For the *bla*_CTX-M_-negative, ceftriaxone nonsusceptible group, a likely mechanism is the presence of ESBLs other than CTX-M, e.g., TEM, SHV, VEB, GES, PER, TLA, BES, and/or SFO enzymes. In addition, p-AmpC production also gives a similar phenotype. Current knowledge on the prevalence of p-ampC is poor because no commercially available diagnostic tests detect its presence. Detection of p-ampC should be considered in future genotypic platforms because 17% of ceftriaxone nonsusceptible Enterobacterales in the United States harbor both ESBL and p-ampC (14). This highlights the limitation of using ceftriaxone resistance as a marker of ESBL production because mechanisms other than ESBL production also confer ceftriaxone resistance. This was observed most with E. cloacae in our study. Using ceftriaxone nonsusceptibility as a marker of ESBL production also encourages carbapenem overuse, and in a small percentage of *bla*_CTX-M_-positive but ceftriaxone-susceptible cases seen in our study, the presence of ESBL goes unrecognized leading to further spread of ESBL (15). Some authors, therefore, favor routine testing of ESBL for therapeutic, infection control, and epidemiological purposes (15). Furthermore, the value of a mechanistic approach to choosing antibiotics is well-demonstrated by the MERINO trial. This trial showed that, despite the result that piperacillin-tazobactam appeared to be active *in vitro* against ceftriaxone non-susceptible isolates, higher mortality of 8.6% was seen in patients treated with piperacillin-tazobactam, compared to the patients treated with meropenem (16). 86% of isolates in this trial were ESBL producers, showing that carbapenems are preferred for the treatment of patients with bacteremia caused by an ESBL producer.

Of note, the 2019 edition of the Clinical and Laboratory Standards Institute document does address reporting of cases with discordance in phenotypic and genotypic results, and recommends repeating the molecular and phenotypic tests, and checking for mixed cultures (4). If the conflict cannot be resolved or explained, then both results should be reported (4).

Our study confirmed the previous findings (3) that, while any Gram-negative species can harbor ESBL-encoding genes, they are most prevalent in K. pneumoniae, E. coli, *and* P. mirabilis. *bla*_CTX-M_ was detected in about a quarter of E. coli and K. pneumoniae isolates in our study, and 16.4% of P. mirabilis isolates. It was also occasionally seen in E. cloacae complex, C. freundii, K. aerogenes, M. morganii, and Salmonella sp.

It is worth noting the limitations of our study. First, we lacked patient-level data. Specifically, it would be pertinent to report management and outcomes of *bla*_CTX-M_-positive ceftriaxone-susceptible cases. This can be a goal for future research in the area. Second, while the protocol only allows ePlex on the first blood culture bottle in a 72h period, we had seen in our experience inadvertent ePlex results that did not fit the prespecified criteria, therefore potentially skewing the reported prevalence of resistance targets. However, the persistence of Gram-negative bacteremia over multiple cultures is less common than for Gram-positive bacteria. Third, further molecular characterization of discordant isolates could provide valuable information and was not performed in the present study. This should be a separate investigation in which a larger collection of discordant isolates should be studied to address the issues discussed above, and the *bla*_CTX-M_ gene in ceftriaxone-susceptible strains should be compared to the *bla*_CTX-M_ gene in ceftriaxone-non-susceptible strains. Fourth, some of these data were included in a recent publication (3). However, this study adds significant data to the previous study because we now report the frequency of blaCTX-M over a longer time, and correlate the results obtained by ePlex to the phenotypic susceptibility profiles obtained by Vitek2 or Microscan, highlighting a clinically important issue of genotypic-phenotypic discordance, which previously has not been studied in detail. Lastly, susceptibility testing in our study was performed by automated systems, which can have limitations. Ideally, in a prospectively done study, these discordant results will be retested by the disk diffusion method. However, because the discordant isolates in our study were highly β-lactam susceptible despite blaCTX-M detection, one would expect disk diffusion to give the same phenotypic results.

In conclusion, we described a series of phenotypic-genotypic discordant isolates. Optimal management of discrepant cases is not defined. Based on this study, we recommend that results from molecular and phenotypic results should be considered in aggregate. Because mechanisms of resistance continue to evolve and diversify, genotypic and phenotypic results both need to be considered in totality for optimal management of patients.

## MATERIALS AND METHODS

### Study sites.

The study was done at the Clinical Microbiology Laboratory (CML) of the Mount Sinai Hospital, New York which processes samples for eight hospitals in the Mount Sinai Health System, including Mount Sinai Beth Israel, Mount Sinai Brooklyn, Mount Sinai Hospital, Mount Sinai Queens, Mount Sinai West, Mount Sinai Morningside, Mount Sinai South Nassau, the New York Eye and Ear Infirmary of Mount Sinai, and associated outpatient facilities.

### Bacteriologic diagnostics and exclusions.

Data from ePlex BCID-GN Panels (GenMark Diagnostics Inc.) performed on blood cultures that signaled positive between March 29, 2019 and February 7, 2021 were retrieved and compared with results of antimicrobial susceptibility testing for Gram-negative bacteria isolated by subculture from the blood culture bottles. Identification of the bacteria is performed by matrix-assisted laser desorption ionization-time of flight mass spectrometry (MALDI-ToF MS).

The ePlex BCID GN panel detects the following resistance genes among Gram-negative bacteria in blood: CTX-M, KPC, OXA-48-like and OXA-23 (a single target), NDM, IMP, and VIM. Some of these data from April 2019 to July 2020 were included in the recent publication by Tamma et al. (3) Gram-positive bacteria and anaerobes were excluded. Also excluded were 13 isolates of Stenotrophomonas maltophilia due to their intrinsic carbapenemases. Results that were positive for more than one Gram-negative bacterial species or that were culture-negative were excluded from frequency analysis with the following exceptions where isolates were retained for analysis. Two isolates were identified as both E. cloacae complex and Enterobacter noncloacae complex by ePlex, but MALDI-ToF MS identified both as E. cloacae. Both these isolates were classified as E. cloacae complex. Two isolates were identified as both K. oxytoca and K. pneumoniae by ePlex but only as K. pneumoniae by MALDI-ToF MS and were classified as K. pneumoniae. One isolate was identified as K. pneumoniae by MALDI-ToF MS and as K. oxytoca by ePlex. Investigators decided to classify this isolate as K. pneumoniae due to our greater clinical experience with MALDI-ToF MS. Lastly, three isolates were identified as K. oxytoca*/*Raoultella ornithinolytica by MALDI-ToF MS but were identified as K. oxytoca by ePlex, and were grouped with K. oxytoca.

Antimicrobial susceptibility testing (AST) was performed using Vitek 2 GN 67 (bioMérieux Clinical Diagnostics; March 2019–December 2020) or Microscan NM 53 (Beckman Coulter Inc.; December 2020–February 2021). Per institutional protocol, AST is not repeated if the same isolate grows in a 72 h period, unless requested by the treating clinician. As a result, not all isolates had AST performed. In addition, ePlex was performed only of the first positive blood culture in the 72 h period per institutional laboratory protocol. ePlex was repeated for subsequent blood cultures if the morphology on a Gram stain was different from the previous Gram stain.

### Data collection and analysis.

The deidentified data set extracted from the CML information systems contained the bacterial identification by ePlex and MALDI-ToF MS, resistance genes identified, the name of the hospital, and the linked susceptibility testing results, if performed on the isolate. Resistant and intermediate categories were grouped into nonsusceptible. For cefepime, a dose-dependent susceptible category was recorded separately. The data were extracted and analyzed on Microsoft Excel 2016. Statistical analysis included only comparisons of categorical variables; therefore, the chi-square test was used for two-by-two comparisons to calculate *P* values. *P* <0.05 was considered statistically significant.

### Ethics statement.

These data were initially reviewed by CML as part of the internal quality improvement project. For this study, the data were deidentified and protected health information removed before analysis. Because this was a laboratory data analysis only and no protected health information was accessed, IRB approval was not needed.

## References

[1] Tamma PD, Aitken SL, Bonomo RA, Mathers AJ, van Duin D, Clancy CJ. 2021. Infectious Diseases Society of America guidance on the treatment of extended-spectrum β-lactamase producing Enterobacterales (ESBL-E), carbapenem-resistant Enterobacterales (CRE), and Pseudomonas aeruginosa with difficult-to-treat resistance (DTR-P aeruginosa). Clin Infect Dis 72:1109–1116. doi:10.1093/cid/ciab295. 33830222

[2] Rossolini GM, D'Andrea MM, Mugnaioli C. 2008. The spread of CTX-M-type extended-spectrum β-lactamases. Clin Microbiol Infect 14:33–41. doi:10.1111/j.1469-0691.2007.01867.x. 18154526

[3] Tamma PD, Smith TT, Adebayo A, Karaba SM, Jacobs E, Wakefield T, Nguyen K, Whitfield NN, Simner PJ. 2021. Prevalence of bla (CTX-M) genes in Gram-Negative bloodstream isolates across 66 hospitals in the United States. J Clin Microbiol 59. doi:10.1128/JCM.00127-21. PMC8316135 33827899

[4] CLSI. 2019. Performance Standards for Antimicrobial Susceptibility Testing. 29th ed CLSI supplement M100. Clinical and Laboratory Standards Institute, Wayne, PA.

[5] Lee N-Y, Lee C-C, Li C-W, Li M-C, Chen P-L, Chang C-M, Ko W-C. 2015. Cefepime therapy for monomicrobial Enterobacter cloacae bacteremia: unfavorable outcomes in patients infected by cefepime-susceptible dose-dependent isolates. Antimicrob Agents Chemother 59:7558–7563. doi:10.1128/AAC.01477-15. 26416853 PMC4649147

[6] Yee R, Dien Bard J, Simner PJ. 2021. The genotype-to-phenotype dilemma: how should laboratories approach discordant susceptibility results? J Clin Microbiol 59:e00138-20. doi:10.1128/JCM.00138-20. PMC8316082 33441396

[7] Zeynudin A, Pritsch M, Schubert S, Messerer M, Liegl G, Hoelscher M, Belachew T, Wieser A. 2018. Prevalence and antibiotic susceptibility pattern of CTX-M type extended-spectrum β-lactamases among clinical isolates of gram-negative bacilli in Jimma, Ethiopia. BMC Infect Dis 18:524. doi:10.1186/s12879-018-3436-7. 30342476 PMC6196031

[8] Hirvonen VHA, Spencer J, van der Kamp MW. 2021. Antimicrobial resistance conferred by OXA-48 β-Lactamases: towards a detailed mechanistic understanding. Antimicrob Agents Chemother 65:e00184-21. doi:10.1128/AAC.00184-21. 33753332 PMC8316048

[9] Djamdjian L, Naas T, Tandé D, Cuzon G, Hanrotel-Saliou C, Nordmann P. 2011. CTX-M-93, a CTX-M variant lacking penicillin hydrolytic activity. Antimicrob Agents Chemother 55:1861-6. doi:10.1128/AAC.01656-10. 21343457 PMC3088237

[10] Schneider I, Queenan AM, Markovska R, Markova B, Keuleyan E, Bauernfeind A. 2009. New variant of CTX-M-type extended-spectrum beta-lactamases, CTX-M-71, with a Gly238Cys substitution in a Klebsiella pneumoniae isolate from Bulgaria. Antimicrob Agents Chemother 53:4518–4521. doi:10.1128/AAC.00461-09. 19620330 PMC2764165

[11] Salimnia H, Veltman J, Chandrasekar PH, Pogue JM, Mynatt R, Salimnia T, Marshall SH, Hujer AM, Bonomo RA. 2020. Carbapenem-susceptible Klebsiella pneumoniae and Escherichia coli isolates carrying a truncated KPC carbapenemase: a challenge for rapid molecular diagnostics. J Clin Microbiol 58:e01627-19. doi:10.1128/JCM.01627-19. 31852760 PMC7041582

[12] Naas T, Oueslati S, Bonnin RA, Dabos ML, Zavala A, Dortet L, Retailleau P, Iorga BI. 2017. Beta-Lactamase DataBase (BLDB) – structure and function. J Enzyme Inhib Med Chem 32:917–919. doi:10.1080/14756366.2017.1344235. 28719998 PMC6445328

[13] Ghiglione B, Rodríguez MM, Curto L, Brunetti F, Dropa M, Bonomo RA, Power P, Gutkind G. 2018. Defining substrate specificity in the CTX-M Family: the role of Asp240 in ceftazidime hydrolysis. Antimicrob Agents Chemother 62:e00116-18. doi:10.1128/AAC.00116-18. 29632016 PMC5971610

[14] Bhalodi AA, Magnano P, Humphries RM. 2020. Performance of ceftriaxone susceptibility testing on the Accelerate Pheno system of ESBL-producing isolates. Diagn Microbiol Infect Dis 98:115171. doi:10.1016/j.diagmicrobio.2020.115171. 32927411

[15] Tamma PD, Humphries RM. 2021. PRO: testing for ESBL production is necessary for ceftriaxone-non-susceptible Enterobacterales: perfect should not be the enemy of progress. JAC-Antimicrobial Resistance 3:dlab019. doi:10.1093/jacamr/dlab019. 33987537 PMC8103002

[16] Harris PNA, Tambyah PA, Lye DC, Mo Y, Lee TH, Yilmaz M, Alenazi TH, Arabi Y, Falcone M, Bassetti M, Righi E, Rogers BA, Kanj S, Bhally H, Iredell J, Mendelson M, Boyles TH, Looke D, Miyakis S, Walls G, Al Khamis M, Zikri A, Crowe A, Ingram P, Daneman N, Griffin P, Athan E, Lorenc P, Baker P, Roberts L, Beatson SA, Peleg AY, Harris-Brown T, Paterson DL, MERINO Trial Investigators and the Australasian Society for Infectious Disease Clinical Research Network (ASID-CRN). 2018. Effect of piperacillin-tazobactam vs meropenem on 30-day mortality for patients with E coli or Klebsiella pneumoniae bloodstream infection and ceftriaxone resistance: a randomized clinical trial. JAMA 320:984–994. doi:10.1001/jama.2018.12163. 30208454 PMC6143100

